# A novel heterozygous HTRA1 mutation is associated with autosomal dominant hereditary cerebral small vessel disease

**DOI:** 10.1002/mgg3.1111

**Published:** 2020-04-02

**Authors:** Zhong‐ling Zhuo, Lu Cong, Jun Zhang, Xiao‐tao Zhao

**Affiliations:** ^1^ Department of Clinical Laboratory Peking University People's Hospital Beijing China; ^2^ Department of Neurology Peking University People's Hospital Beijing China

**Keywords:** autosomal dominant hereditary cerebral small vessel disease, heterozygous mutation, HTRA1, TGF‐β1/Smad signaling, whole‐exome enrichment and sequencing

## Abstract

**Background:**

We investigated whether a heterozygous mutation that we newly identified in *HTRA1* (high‐temperature requirement serine protease A1 gene) in a pedigree with autosomal dominant hereditary cerebral small vessel disease (SVD) reduces the function of HTRA1 and affects the transforming growth factor‐β1 (TGF‐β1)/Smad signaling.

**Methods:**

Whole‐exome sequence from the proband and her two sisters was examined using whole‐exome enrichment and sequencing. Expression of HTRA1 and TGF‐β1/Smad and HTRA1 activity were assayed using sodium dodecyl sulfate‐polyacrylamide gel electrophoresis and western blotting analyses after transfecting wild‐type and mutant HTRA1 genes into HEK293 cells.

**Results:**

A new heterozygous mutation (c.614C>G:p.Ser205Cys) in *HTRA1* was identified in the sequence encoding the trypsin‐like serine protease domain. The mutation was predicted to be deleterious by in silico tools. Moreover, in vitro activity and protein analyses revealed a loss‐of‐function effect of the mutation: the proteolytic activity of mutant HTRA1 was decreased, and, notably, this was accompanied by an increase in TGF‐β1/Smad protein levels.

**Conclusions:**

The heterozygous mutation HTRA1 S205C causing diminished protease activity is associated with—and could represent a cause of—autosomal dominant hereditary cerebral SVD. Our results also indicate a relationship between HTRA1 and TGF‐β1/Smad signaling.

## Graphical abstract

The heterozygous mutation high‐temperature requirement serine protease A1 gene (HTRA1) S205C causing diminished protease activity is associated with—and could represent a cause of—autosomal dominant hereditary cerebral small vessel disease. Our results also indicate a relationship between HTRA1 and transforming growth factor‐β1/Smad signaling.

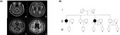

## 1 INTRODUCTION

Cerebral small vessel disease (SVD) represents a group of heterogeneous diseases that affect the veins, small arteries, arterioles, and/or capillaries of the brain (Pantoni, 2010; Wardlaw, Smith, & Dichgans, 2013). Several early onset single‐gene forms of SVD have been reported in adult patients, including cerebral autosomal dominant arterial disease with subcortical infarction and leukoencephalopathy (CADASIL), hereditary cerebral amyloid angiopathies, angiopathies associated with mutations in COL4A1 and COL4A2 genes (Gould et al., 2006; Haffner & Vinters, 2016; Joutel et al., 1996; Rutten‐Jacobs & Rost, 2019), and cerebral autosomal recessive arterial disease with subcortical infarction and leukoencephalopathy (CARASIL). CADASIL is caused by *NOTCH3* mutation and is the most common hereditary SVD in >500 families reported thus far (Chabriat, Joutel, Dichgans, Tournier‐Lasserve, & Bousser, 2009). By contrast, CADASIL type 2 (OMIM# 616779) is an extremely rare autosomal dominant hereditary cerebral SVD, and only a few cases of this disease have been reported (Kono et al., 2018; Wu et al., 2018; Xie & Zhang, 2018); the disease is caused by monoallelic mutation of high‐temperature requirement serine protease A1 gene (*HTRA1*), the gene encoding high‐temperature requirement serine protease A1. HTRA‐family proteins have been reported to downregulate transforming growth factor‐β1 (TGF‐β1)/Smad signaling (Oka, 2004; Tocharus et al., 2004), and HTRA1 gene mutations can reduce protease activity (Verdura et al., 2015) and lead to the upregulation of p‐Smad2/3 levels (Hara et al., 2009). This is regarded as the underlying cause of cerebral arteriolar lesions and extraneurological symptoms. Here, we used whole‐exome enrichment and sequencing to detect the whole‐exome sequence of a proband and her two sisters, and we found that a missense heterozygous mutation (c.614C>G:p.Ser205Cys) in the HTRA1 protease domain in this pedigree with autosomal dominant hereditary cerebral SVD produces inactive HTRA1 protein. We transfected the mutant HTRA1 gene into HEK293 cells and examined TGF‐β1/Smad and HTRA1 expression levels, and we also investigated whether the mutation reduced the proteolytic activity of HTRA1.

## 2 METHODS

### 2.1 Ethics statement

The ethics committee of our hospital approved the protocols of this study, which was conducted according to the Declaration of Helsinki. Written informed consent was obtained from the participants for the publication of the case report.

### 2.2 Whole‐exome enrichment and sequencing

Whole‐exome enrichment and sequencing can be performed using the procedures developed for multiple platforms that are currently available. We adopted the protocol followed by the Agilent SureSelect Human All Exon methodology, which can be used to analyze ~50‐Mb exonic regions. Coding DNA sequences (CDSs) that were captured using ~120‐base RNA probes (SureSelect) were identified from the NCBI Consensus CDS Database and other databases, including Sanger miRBase. The adopted protocols were implemented at the laboratory bench level (automation was not required), and the generated library was used for next‐generation sequencing (performed on an Illumina HiSeq XTM Ten sequencer).

### 2.3 Sanger sequencing

Gene mutations were identified using Sanger sequencing. The following primer sequences were used: HTRA1‐F: GCGTTCATTTTAAGGTGCTACAG; HTRA1‐R: GGCCATACTCAGCATCTCCT.

### 2.4 Assay of HTRA1 function and TGF‐β1/Smad signaling

#### 2.4.1 Plasmid constructs

First, we designed a primer set to synthesize the entire CDS of *HTRA1* (NG_011554.1): HTRA1‐WT‐F: CTTGGTACCGAGCTCGGATCCATGCAGATCCCGCGCGCC; HTRA1‐WT‐R: TGATGGATATCTGCAGAATTCCTATGGGTCAATTTCTTCGGGA. Site‐directed mutagenesis (Invitrogen System) was used to generate the cDNA encoding the mutant form of HTRA1. The cDNAs were successfully subcloned individually into pcDNA3.0 vector (Invitrogen) using a homologous recombination method, which included these steps: (a) linearization of cloning vector; (b) design of fragment‐amplification primer; (c) insertion of PCR‐amplified fragment; (d) recombination reaction; (e) conversion of reaction product, coating; and (f) clone identification.

#### 2.4.2 Transfection and cell culture

HEK293 cells were cultured at 37°C and 5% CO_2_ in Dulbecco's Modified Eagle Medium containing glucose at high concentration, 10% fetal bovine serum, 100 μg/ml streptomycin, and 100 U/ml penicillin (all from Life Technologies). Plasmids were transiently transfected into cells using Lipofectamine 3000, and treated cells were harvested after 48 hr of culture. These experiments were repeated at least thrice.

#### 2.4.3 Western blotting

Proteins were separated using SDS‐PAGE and transferred onto polyvinylidene fluoride or polyvinylidene difluoride membranes (Millipore), which were blocked in 5% skim milk and then probed with primary antibodies: HTRA1 rabbit mAb (ab199529; Abcam), Smad2/3 rabbit mAb (8685S; Cell Signaling Technology [CST]), Smad4 rabbit mAb (38454S; CST), Phospho‐Smad2 (Ser465/467)/Smad3 (Ser423/425) rabbit mAb (8828S; CST), or GAPDH mouse mAb (sc47724; Santa Cruz Biotechnology). Immunoreactive bands were detected using horseradish peroxidase‐conjugated goat anti‐rabbit or anti‐mouse IgG (ZSGB‐BIO, ZB‐5301 or ZB‐5305). All experiments were repeated in triplicate.

#### 2.4.4 HTRA1 activity assays

Bovine serum albumin (BSA; 0.4 mg/ml) was denatured using 1.5‐mmol/L dithiothreitol and then exposed (at 37°C for 24 hr) to conditioned medium from cultured HEK293 cells transfected with wild‐type (WT) or mutant HTRA1 plasmid. Bovine serum albumin degradation was assessed using SDS‐PAGE and Coomassie Brilliant Blue staining. These experiments were repeated in triplicate.

### 2.5 Statistical analysis

All data were analyzed using one‐way ANOVA and are presented as means ± *SD*. Differences were considered significant at *p* < .05 (*p < .05; **p < .01). All statistical analyses were performed using SPSS version 19.0 software.

## 3 RESULTS

### 3.1 Case report

A 53‐year‐old female proband (II: 5; Figure 1B) experienced cognitive slowing without marked impact on daily activities starting from age 46, and progressive reduction in cognitive performance and recurrent minor strokes developed in the next 3 years. At age 50, the patient experienced an acute episode of ischemic stroke of the lacunar type that led to right limb hemiparesis. Further deterioration of her disease presented as forced crying and laughing at age 53. The patient's Mini‐Mental State Examination score for cognitive testing was 17/30. A follow‐up brain magnetic resonance imaging (MRI) of the proband (II: 5; Figure 1A) revealed diffuse leukoencephalopathy in T2‐weighted images, and white matter hyperintensities in the periventricular region and multiple lacunar infarcts were also detected, predominantly in the basal ganglia and brainstem. The proband's 60‐year‐old sister (II: 1; Figure 1B) had a history of low back pain and headache; in 2005, when she was 47 years old, she experienced recurrent left limb weakness and dysarthria. At age 52, she presented a low mood, and, subsequently, she presented cognitive complaints and progressive impairment in motor functions. The sister's brain MRI results were in several respects similar to those of the proband (Figure 2). T2‐weighted and fluid‐attenuated inversion recovery MRI disclosed diffuse leukoencephalopathy and lacunas. The proband and her sister had no previous history of classic vascular risk factors or symptoms of alopecia. Their mother (I: 2) had a similar neurological history as the proband, and presented with recurrent ischemic strokes and cognitive decline at age 60. Other members of the family, including a brother and a sister of the proband, were not clinically affected.

**Figure 1:**
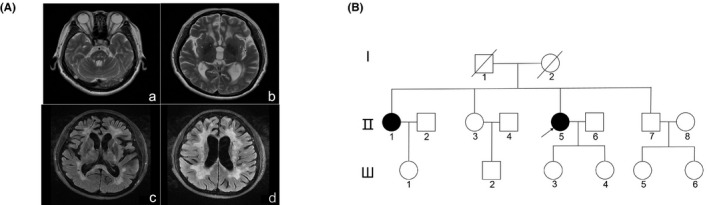
Brain magnetic resonance imaging (MRI), pedigree, and gene analysis of proband. (A) Brain MRI of proband: T2‐weighted images (a, b) showing multiple lacunar infarcts in the pons and basal ganglion; fluid‐attenuated inversion recovery MRI (c, d) showing diffuse leukoencephalopathy involving the periventricular region. (B) Pedigree of family with autosomal dominant hereditary cerebral small vessel disease. Arrow: proband; solid symbols: patients with autosomal dominant hereditary cerebral small vessel disease; symbol with slash: deceased person

**Figure 2:**
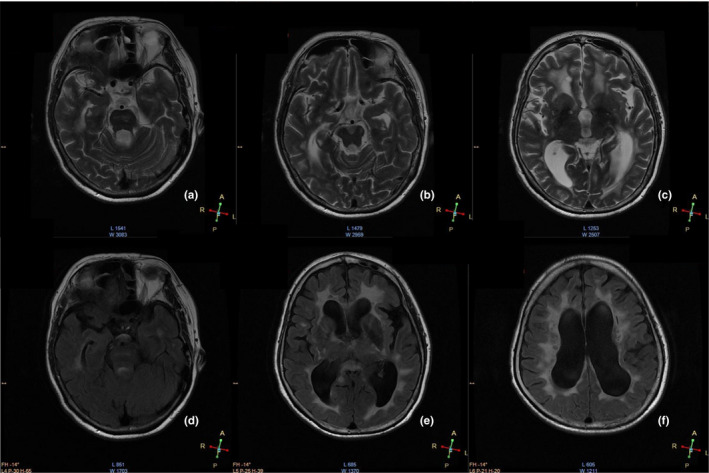
Brain magnetic resonance imaging (MRI) of 60‐year‐old sister of the proband. Brain MRI of proband's sister: White matter hyperintensities in temporal lobe and periventricular region in T2‐weighted MRI (a–c) and fluid‐attenuated inversion recovery MRI (d–f); diffuse cortical and subcortical white matter atrophy and multiple lacunar infarcts in the pons, cerebral peduncle, and basal ganglion are observed

### 3.2 Identification and characterization of *HTRA1* mutation

**Figure 3:**
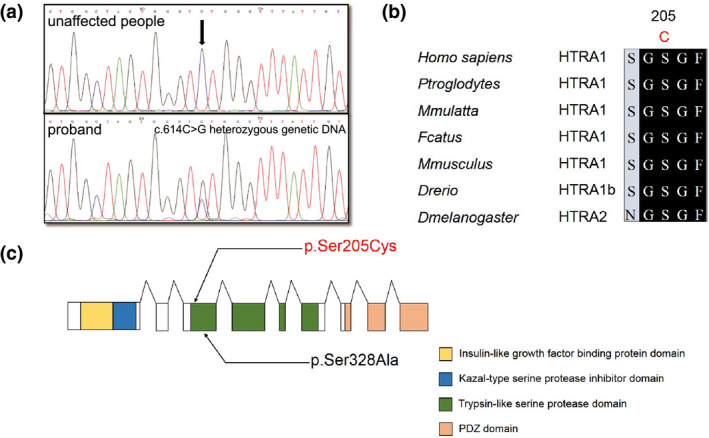
High‐temperature requirement serine protease A1 gene (HTRA1) S205C mutation in autosomal dominant hereditary cerebral small vessel disease. (a) Whole‐exome mutation analysis in proband, identifying a new heterozygous mutation in exon 3 of *HTRA1* (c.614C>G, p.Ser205Cys). (b) Distribution of mutations in *HTRA1*, which contains nine exons (squares) encoding an insulin‐like growth factor binding protein domain (yellow), a Kazal‐type serine protease inhibitor domain (blue), a trypsin‐like serine protease domain (green), and PDZ domain (orange). Nucleotide and corresponding amino acid mutations are listed: S205C in red, S328A in black. (c) Conservation of mutated HTRA1 amino acid residues in patients with CARASIL and in nonhuman species. Conserved residues are shaded thus: black, 100% conserved; dark gray, 80% conserved. The C at position 205 is highlighted in red; mutations at this position are either completely or largely conserved. Sequences were obtained from GenBank

According to the clinical results and white matter lesions, we first considered the hypothesis of hereditary SVD. We performed whole‐exome screening of genes, including *HTRA1* and *NOTCH3*, to distinguish CARASIL, CADASIL, autosomal dominant hereditary cerebral SVD, and other related diseases. Whole‐exome mutation screening of blood samples from the proband (II: 5) and her sister (II: 1) revealed an unusual heterozygous missense variant in exon 3 (c.614C>G) in *HTRA1* (Figure 3a). This mutation is unlikely to result from gene polymorphism, because it has not been reported in SNP databases or the 1000 Genomes database, and it was not detected in other chromosomes that serve as controls (Figure 3b). Furthermore, this mutation is positioned in the region that encodes the serine protease domain (204–364 aa) of HTRA1 (Figure 3c). In this domain, S328A substitution was shown to abolish HTRA1 protease activity (Hu et al., 1998). The HTRA1 mutation identified here generates a missense variant with the amino acid substitution p.Ser205Cys, which was predicted to be damaging by PolyPhen2 (genetics.bwh.harvard.edu/pph), sorting intolerant from tolerant (SIFT) (sift.jcvi.org), and MutationTaster (mutationtaster.org). The identical heterozygous p.Ser205Cys mutation was detected in the proband (II: 5), her sister (II: 2), her unaffected daughter (Ш: 4), and her sister's unaffected daughter (Ш: 1; Figure 1B).

### 3.3 Expression of mutant HTRA1

**Figure 4:**
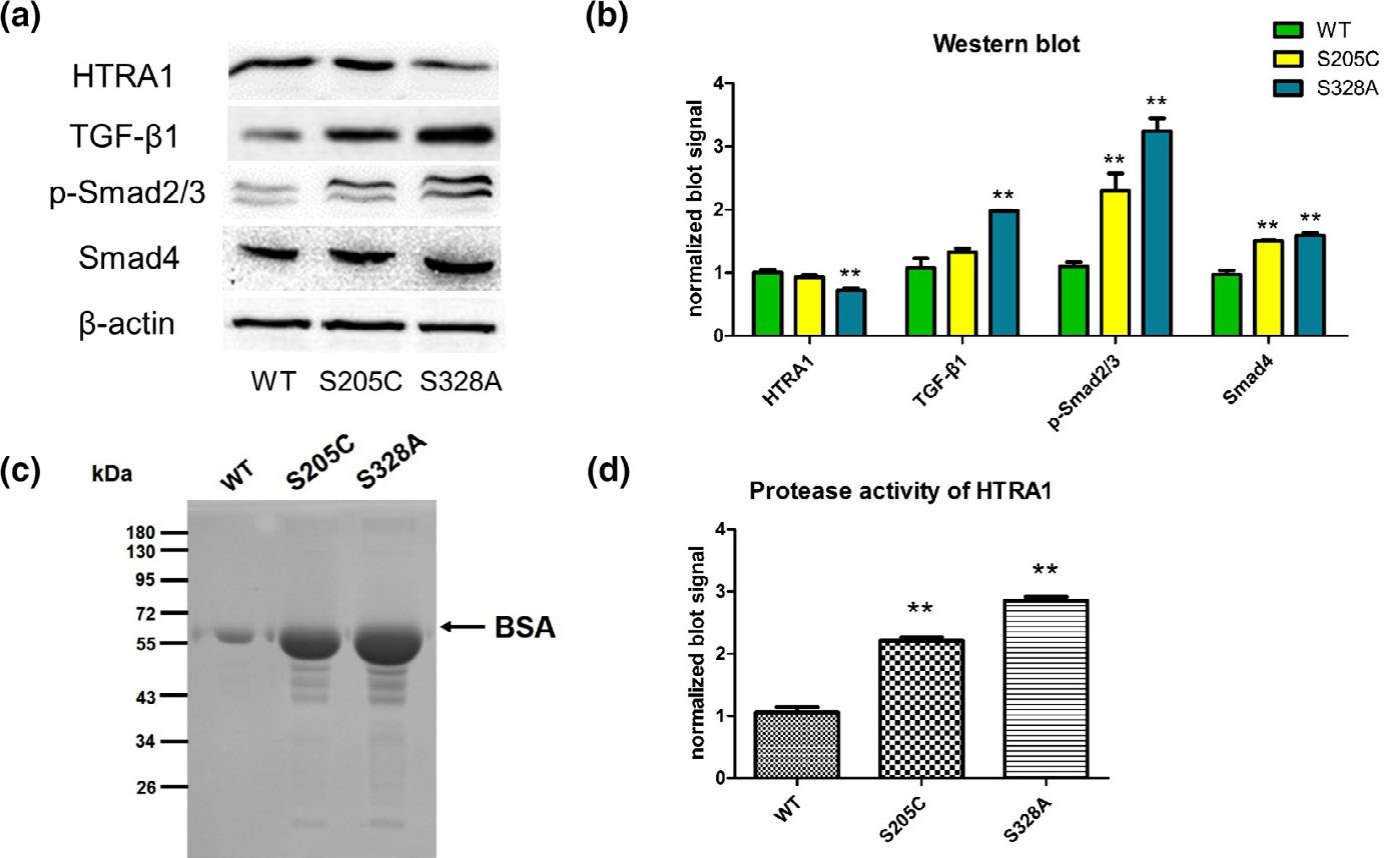
Impact of high‐temperature requirement serine protease A1 gene (HTRA1) mutation on transforming growth factor‐β1 signaling in HEK293 cells. (a) Western blotting of extracts of HEK293 cells transfected with plasmids encoding wild‐type, S205C, and S328A HTRA1. (b) Differences in protein levels, compared using one‐way ANOVA. (c) Proteolytic activity of conditioned media toward denatured bovine serum albumin, evaluated through SDS‐PAGE and Coomassie Blue staining. (d) Differences in proteolytic activity, compared using one‐way ANOVA

To investigate HTRA1 mRNA and protein expression, WT and mutant HTRA1 genes were transfected into HEK293 cells; for this analysis, we constructed pcDNA3.0 carrying WT HTRA1 gene, S205C mutant HTRA1 gene, or S328A mutant HTRA1 gene. The active‐site mutant, S328A, was used as the positive control (Hu et al., 1998). HTRA1 gene expression was detected in all groups, and western blotting results showed that HTRA1 expression was lower in the S205C and S328A groups than in the WT group (*p* = .002; Figure 4a,b).

### 3.4 Mutant HTRA1 inhibits TGF‐β1/Smad signaling

Next, we examined the potential inhibitory effect of the HTRA1 mutation on TGF‐β1/Smad signaling. To investigate the pathway through which TGF‐β1/Smad signaling might be blocked, we measured the degree of phosphorylation of Smads, which are downstream effectors of the TGF‐β1 family. We prepared protein extracts from the three groups of cells (WT, S205C, and S328A) at 48 hr after transfection and used western blotting to detect Smad2/3, Smad4, phosphorylated Smad2/3, and TGF‐β1: The levels of Smad2/3, Smad4, phosphorylated Smad2/3, and TGF‐β1 and those of Smad2/3 and phosphorylated Smad2/3 were higher in the S205C and S328A groups, respectively, than in the WT group (*p* < .01), although the slight increase in TGF‐β1 in the S205C group was not statistically significant (Figure 4a,b).

### 3.5 Protease activity of mutant HTRA1

High‐temperature requirement serine protease A1 gene encodes a conserved serine protease domain (Figure 3c; Clausen, Southan, & Ehrmann, 2002), and mutations in this domain markedly reduce HTRA1 proteolytic activity (Nozaki, Nishizawa, & Onodera, 2014). Therefore, we used the transiently transfected HEK293 cells and BSA substrate for in vitro assessment of HTRA1 catalytic activity in culture supernatants (conditioned media), with the S328A mutant used as the control: the activity of HTRA1 S205C mutant was significantly diminished (*p* < .01) relative to WT and the reduction in activity was similar to that in the S328A mutant (Figure 4c,d).

## 4 DISCUSSION

Because white matter lesions were detected in MRI scans, CADASIL was initially diagnosed in our proband. However, the detection of the novel heterozygous mutation in exon 3 (p.Ser205Cys) of *HTRA1* in the proband and other members in this pedigree excluded CADASIL, for which *NOTCH3* at 19p13 has been identified as the only causative gene. CARASIL is associated with mutations in *HTRA1*, but the characteristic extraneurologic symptoms of CARASIL, including alopecia, were lacking in this pedigree; more importantly, the genetic pattern in this pedigree did not conform to autosomal recessive inheritance. We therefore doubted a diagnosis of CARASIL. Intriguingly, Verdura and coworkers (Verdura et al., 2015) had reported that heterozygous *HTRA1* mutations are associated with autosomal dominant hereditary cerebral SVD.

The most unexpected finding of this study is the deleterious role of heterozygous mutations in *HTRA1*; the following evidence supports the deleterious effect of the mutation identified here. First, the mutation was localized in the *HTRA1* region that encodes the serine protease domain (Figure 3c), in which the S328A substitution was previously shown to abrogate protease activity (Hu et al., 1998). Notably, the mutation studied here produces a missense variant with the p.Ser205Cys substitution, which was predicted to be deleterious by both PolyPhen2 and SIFT. Second, the HTRA1 mutation resulted in diminished levels of HTRA1 proteolytic activity and elevated levels of TGF‐β1/Smad in HEK293 cells. This agrees with a previous report (Hara et al., 2009) that missense mutations and one of the nonsense mutations in the gene generated protein products that exhibited low levels of protease activity and did not repress signaling by the TGF‐β family, but that the other nonsense mutation resulted in the loss of HTRA1 protein through nonsense‐mediated decay of mRNA. We believe that this mechanism might lead to stenosis of cerebral arterioles, clinical symptoms of the nervous system, and the corresponding appearance of a wide range of leukoencephalopathies.

High‐temperature requirement serine protease A1 gene, the first human HTRA‐family protein to be cloned and sequenced, is derived from an open reading frame encoding a 480‐aa protein that contains a N‐terminal domain including a signal peptide (codons 1–22), an IGFBP domain (codons 33–100), a Kazal inhibition region (codons 101–155), and the region that confers the hydrolase activity of HTRA1 and features a structure composed of three key amino acids: serine, histidine, and aspartic acid. Site‐directed mutagenesis of this serine to alanine (S328A) completely deactivates the enzyme (Hu et al., 1998). TGF‐β1/Smads are involved in fundamental intracellular signaling pathways, and the basic signal transduction process in these pathways is this: target binding results in the phosphorylation of TGF‐βRII, which, in turn, leads to the activation of TGF‐βRI, which induces Smad2/3 phosphorylation and heterotrimeric complex formation with Smad4; this complex translocates to the nucleus and regulates the expression of target genes (Yamamoto & Ihara, 2017). Because Smad2 and Smad3 and their phosphorylated forms are crucial players in the signaling pathway and Smad4 plays a “central” role, we selected these markers to test for potential changes in TGF‐β1/Smad signaling following HTRA1 mutation.

Transforming growth factor‐β1 is the target protein for HTRA1, a serine protease widely integrated into the TGF‐β1 protein family. In a previous study (Campioni et al., 2011) that isolated 13 HTRA1‐interacting proteins in vitro and in vivo using yeast two‐hybrid screening, a key protein was found to be the signaling molecule TGF‐β1. Importantly, disease association with the TGF‐β1/Smad pathway has been demonstrated (Hara et al., 2009). High‐temperature requirement serine protease A1 gene mutations lead to diminished expression or lack of function of HTRA1, which is then unable to block the TGF‐β1/Smad signaling pathway; this ultimately results in expression changes in downstream signaling molecules and target proteins. Verdura et al. (2015) reported that in 10/201 probands (4.97%) with cerebral SVD who carried a heterozygous *HTRA1* mutation, the mutation was predicted to be destructive, and in vitro activity analysis of the HTRA1 mutants demonstrated a loss‐of‐function effect. Several heterozygous HTRA1 mutations were also reported by other investigators (Tateoka et al., 2016; Di Donato et al., 2017; Zhang, Xie, & Lu, 2018; Wu et al., 2018; Kono et al., 2018; Xie & Zhang, 2018), but the relationship between these mutations and TGF‐β signaling was not examined. However, Verdura et al. (2015) used expression vectors in their study to demonstrate that HTRA1 heterozygous mutations resulted in decreased protein activity (Beaufort et al., 2014), and Oka (2004) and Launay et al. (2008) showed that reduced HTRA1 activity led to elevated levels of TGF‐β1/Smads. Therefore, we suspected that the pathogenesis caused by HTRA1 heterozygous mutations is similar to that resulting from HTRA1 homozygous mutations, and we confirmed this in our experiments.

The precise mechanism by which HTRA1 inhibits TGF‐β1/Smad signaling remains unclear. High‐temperature requirement serine protease A1 gene was previously reported to inhibit the TGF‐β1 pathway by removing mature TGF‐β1 extracellularly (Canfield, Hadfield, Rock, Wylie, & Wilkinson, 2007; Gilicze et al., 2007); however, this finding remains debated, and according to the results of an experimental study, HTRA1 inhibits the TGF‐β1 pathway by clearing the precursor protein in the endoplasmic reticulum and the cleared product is degraded by the endoplasmic reticulum‐associated degradation system, which causes a reduction in the maturation of TGF‐β1 (Launay et al., 2008). Besides regulating target‐gene transcription through the Smad family, TGF‐β1 can influence the transcription of downstream genes by acting on other signaling pathways, such as through activation of the small GTP‐binding proteins Ras and Rho. Considerable research attention is currently focused on the interactions between TGF‐β1/Smads and other signaling pathways. However, the role of the cross talk between TGF‐β1/Smad signaling pathways in the pathogenesis of autosomal dominant hereditary cerebral SVD is unknown and warrants further investigation. Although very limited research has thus far addressed HTRA1 protein functions and signaling pathways and the ECM in autosomal dominant hereditary cerebral SVD, the study of HTRA1 and its related signaling pathways could produce a breakthrough for future treatment; this is because, TGF‐β1 expression can be inhibited by the antihypertensive drug class of angiotensin I receptor antagonists. These drugs alleviate disease symptoms in an animal model of Marfan syndrome and, notably, in patients with a marked effect on human brain functions. Thus, the application of TGF‐β1 antagonists might represent a novel treatment for autosomal dominant hereditary cerebral SVD and a promising avenue for future research.

## CONFLICT OF INTEREST

None declared.

## AUTHOR CONTRIBUTIONS

ZZ‐Designed and conceptualized study; analyzed the data; drafted the manuscript. LC‐ Designed and conceptualized study; analyzed the data; drafted the manuscript. JZ‐supervised this work, revised the manuscript. XZ‐supervised this work, revised the manuscript.
